# Anabolic Steroid-Induced Myocardial Infarction in a Young Male

**DOI:** 10.7759/cureus.13054

**Published:** 2021-02-01

**Authors:** Fnu Samreen, Ubaidullah Popal, Zulfiqar A Qutrio Baloch

**Affiliations:** 1 Cardiology, National Institute of Cardiovascular Diseases, Karachi, PAK; 2 Internal Medicine, Tabba Heart Institute, Karachi, PAK; 3 Cardiology, Sparrow Hospital, Lansing, USA

**Keywords:** anabolic steroid abuse, myocardial infarction, st-elevation myocardial infarction

## Abstract

Misuse of androgenic-anabolic steroids (AAS) has been well known to increase the risk for a cardiac problem, including acute myocardial infarction (MI). Steroids once thought a magic drug providing immediate relief to patients, also have a darker aspect of its severe side effects. AAS are widely used these days, especially in teenagers, bodybuilders, and athletes. MI is thought to be a disease of old age, but young patients with MI without risk factors draw attention to the possibility of drugs such as cocaine, AAS abuse, and amphetamine.

In this article, we report the case of a 38-year-old African-American male, with a history of AAS abuse, who arrived at the emergency department with complaints of severe chest pain radiating to the left arm. An electrocardiogram (ECG) revealed ST-elevation MI (STEMI) and elevated troponin. The patient was transferred to the cardiac catheterization lab for an emergent catheterization which showed 100% stenosis of the left anterior descending artery and a drug-eluting stent was placed. An echocardiogram showed an ejection fraction of 35%. All blood workup was negative. The patient was discharged on aspirin, ticagrelor, statin, ACE inhibitor, and B-blocker after three days.

Chest pain in a young patient population secondary to MI is not uncommon these days and the most important thing to evaluate is drug history, including AAS use. Athletes, bodybuilders, and others who use steroids or other drugs that are responsible for MI should be under the supervision of physicians so that the complications of steroids are ascertained, and if steroids are needed for any medical illness, proper dosage and follow-up should be emphasized. Therefore, while taking history from a patient, it is essential for physicians to be aware of this association of steroids with coronary artery disease.

## Introduction

Risk factors for myocardial infarction (MI) in young people are highly significant and at this age drug abuse must always be considered. Athletes use androgenic-anabolic steroids (AAS) to increase their performance (protein synthesis in skeletal muscles is increased by AAS); however, there are critical adverse effects, including hepatic and endocrine dysfunction, and cardiovascular and behavioral changes have been reported [1].

Steroids have a broad spectrum of applications, ranging from treatment in medical emergencies and other diseases to abuse by athletes and other sports players. No doubt steroids have many good applications, but its overuse leads to serious consequences and sometimes even death [2].

An increased cardiovascular risk in those individuals who use these drugs has been shown [3]. ST-elevation MI (STEMI) is a life-threatening condition having 2.5-10% mortality in the first month [4]. Conventional risk factors play an important role in coronary heart diseases, and nontraditional risk factors also need to be considered as they are present in more than 50% of coronary artery disease cases [5]. In a previous study, more than 150 drugs were reported as possible causes of MI, out of which 39 drugs were thought as the main suspects that can cause MI: prednisone, betamethasone, and dexamethasone [6].

## Case presentation

A 38-year-old African American male with no significant past medical history (he was using anabolic steroid [unknown duration] for muscle building and athlete) came to the emergency department with complaints of severe chest pain that started 30 minutes previously, which were crushing in nature, radiating to his left arm, and associated with sweating, nausea, and breathlessness. He denied similar pain in the past during exertion or rest. The patient smoked three cigarettes per day for 10 years. His family history was nonsignificant for ischemic heart disease. 

**Figure 1:**
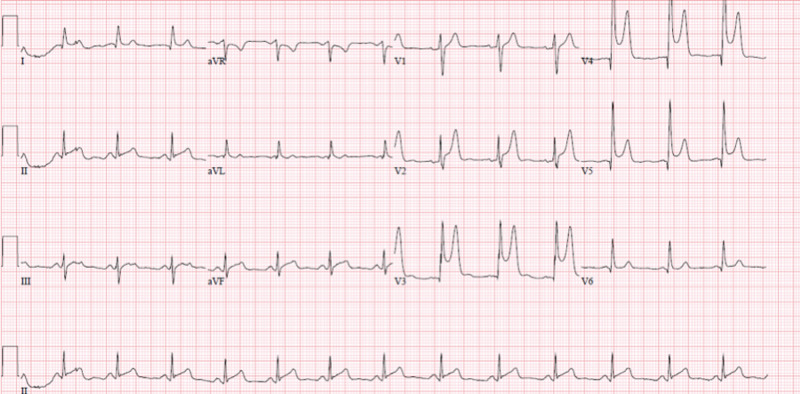
Electrocardiogram ST-elevation in precordial leads V2, V3, V4, and V5.

On examination, the patient was sweaty, and vitals were: heart rate, 110 beats/min; blood pressure, 130/85 mm Hg; respiratory rate, 16/min; temp., 98.6 F [Normal 97-98 F]. His heart sounds were normal with no murmur. There was no lower extremity edema and normal peripheral pulses. A 12-lead electrocardiogram (ECG) showed ST-segment changes of the precordial leads of an acute anterior wall MI (Figure 1). The patient’s serum creatine kinase and total cholesterol concentration were normal. Complete blood count, urea, creatinine, urine analysis, and urine drug screen were normal.

**Figure 2:**
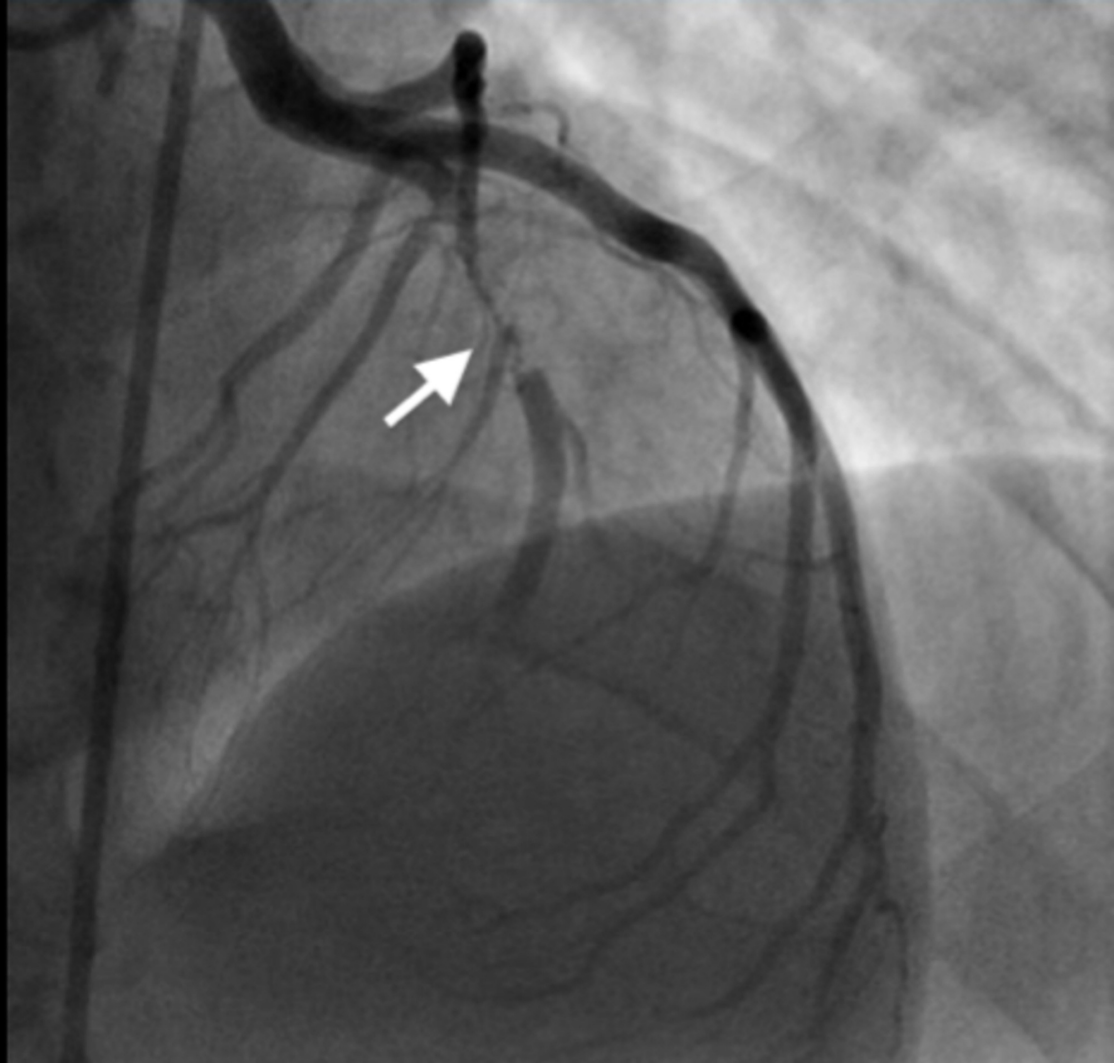
Cardiac catheterization Proximal left anterior descending artery 100% stenosis.

The ECG taken at the time of arrival in the emergency department revealed STEMI and elevated troponin. Cardiac catheterization revealed proximal left anterior descending artery 100% stenosis (Figure 2). A thrombectomy, percutaneous transluminal coronary angioplasty, and drug-eluting stent placement in the left anterior descending artery were performed. An ECG showed ischemic cardiomyopathy left ventricular ejection fraction 35-40%, and apical and anterolateral wall hypokinesis. Systolic function was moderately reduced. There was mild hypokinesis of the mid-anteroseptal and mid-anteroseptal wall. There was akinesis of the apical anterior, apical septal, apical lateral, and the apical wall(s). Doppler parameters were consistent with abnormal left ventricular relaxation (grade 1 diastolic dysfunction).

All blood work including complete blood counts, chemistry, creatinine, liver enzymes, creatine kinase, homocysteine, vitamin B12, TSH, urine analysis, and urine drug screen was negative. The low-density lipoprotein (LDL) level 116 mg/dl, high-density lipoprotein (HDL) 48 mg/dl, triglycerides 141 mg/dl, and total cholesterol 151 mg/dl. The patient’s coagulative workup including antithrombin III, factor V Leiden deficiency, protein C and S activity were within the normal range. Autoimmune workup was checked to rule out vasculitis including antinuclear antibody, antineutrophil cytoplasmic antibody, and anti-glomerular basement membrane antibody, and was negative. 

The patient was stable during the hospital stay and asymptomatic. He was discharged on the third day of hospitalization. He was prescribed aspirin 81 mg daily, ticagrelor 90 mg daily, carvedilol 3.25 mg twice daily, lisinopril 2.5 mg daily, and atorvastatin 80 mg daily. The aldosterone antagonist was not started inpatient secondary to borderline soft blood pressure. The patient was followed up as an outpatient in a week and his blood pressure was within normal limit, he was started on low-dose spironolactone 25 mg, and repeated basic metabolic was checked for potassium and that was within normal limit. The patient also had an echocardiogram after 40 days and his ejection fraction improved, and spironolactone was discontinued.

## Discussion

Bodybuilders, weightlifters, and athletes frequently take AAS, a synthetic derivative of testosterone, in order to increase stamina and muscle mass [7]. Attaining a 10-20% increase in strength of normal muscle mass is the main reason for abuse. Besides this positive aspect, there are a wide range of side effects, involving cardiovascular, cerebrovascular systems, and many more. The pathophysiology behind this is still unclear, but some hypotheses have been proposed. The prothrombotic effect of AAS is thought to be one mechanism. Steroids increase the synthesis of thromboxane A2 and decrease the synthesis of prostacyclin, thus leading to increased platelet aggregation. Also, increased thrombin activity contributes to a hypercoagulable state [3]. In addition, other hypothetical effects have been proposed such as impaired endothelial function and vasospasm. Steroid effects on homocysteine level causing hyperhomocysteinemia can lead to atherosclerotic and thrombotic effects in athletes. AAS can affect the absorption of B6 and B12 vitamins and cause an elevation in homocysteine levels [8].

Dyslipidemia and reduction in antioxidant levels are also reported as possible mechanisms. In a literature review, it was concluded that AAS have multiple effects on the cardiovascular system. Taking above the normal doses of AAS affect the heart structurally as well as functionally. The changes include myocardial hypertrophy, an increase in the diameters of heart chambers, and also marked changes in heart relaxation and contractile function. A temporary rise in blood pressure, prothrombotic effects, impaired lipid metabolism causing increase LDL and decrease HDL result in an increased risk of coronary artery disease. Ultimately AAS abusers can be at increased risk of life-threatening arrhythmias, leading to sudden cardiac death [3].

Athletes abusing AAS frequently attribute their symptoms to their workout and neglect minor symptoms of coronary artery disease and thus when they present to ER, they present with complications.

## Conclusions

The young patient on anabolic steroid who presented to ED secondary to chest pain, such patients always need to work up for coronary artery disease. Athletes abusing AAS frequently attribute their symptoms to their workout and neglect minor symptoms of coronary artery disease and thus when they present to ER, they present with complications.
